# Comment on: “Transversus Thoracic Muscle Plane Block for Attenuating the Haemodynamic Response to Median Sternotomy”

**DOI:** 10.4274/TJAR.2023.221172

**Published:** 2023-08-18

**Authors:** Raghuraman M. Sethuraman

**Affiliations:** 1 Department of Anaesthesiology, Sree Balaji Medical College & Hospital, Chennai, India

**Keywords:** Cardiac surgeries, cardiovascular and thoracic anaesthesia, regional anaesthesia, sternotomy, transversus thoracic muscle plane block

**Dear Editor,**

I read with great interest the case series that evaluated the haemodynamic responses to sternotomy in patients in whom bilateral transversus thoracic muscle plane block (TTPB) was administered.^1^ I wish to present my reflections on that article.

Walian et al.^1^ quoted 3 references from Ueshima et al. (Reference #8-10 of Walian et al.^1^) that were retracted. I believe that these 3 references were cited despite knowing that these referenced articles were retracted because the titles of these references contain “RETRACTED” in bold format. I believe that we are not supposed to cite the retracted articles knowingly and it is the primary responsibility of the authors to be vigilant in avoiding the citation of retracted articles at any stage of publication. Walian et al.^1^ could have deleted these 3 articles by Ueshima et al. during the proof check as these were retracted well ahead on the basis of misconduct, and there are other articles by Fujii et al.^2^ and Zhang et al.^3^ on this topic. Hence, there is no justification to cite these retracted articles. In this context, I wish to point out that recently, the inclusion of a retracted article in a meta-analysis was noted during the production, and remedial measures were taken in the nick of time.^4^ Walian et al.^1^ also misquoted the article by Taketa et al.^5^ for TTPB, while that case series was about erector spinae plane block in thoracoscopic lobectomies; hence, it had nothing to do with TTPB or sternotomy pain.

Walian et al.^1^ stated that their case series was unique as it analysed the role of TTPB specifically on haemodynamic responses. However, although it is technically correct, it is not a great difference considering the previous studies focusing on postoperative pain and analgesic consumption,^2,3^ as sternotomy pain is the major contributing factor in the postoperative phase. Also, the variations in haemodynamic parameters could be influenced by other factors such as volume status, depth of anaesthesia, etc.
