# Supplementary material for: Comment on: “Transversus Thoracic Muscle Plane Block for Attenuating the Haemodynamic Response to Median Sternotomy”
Source: Turk J Anaesthesiol Reanim. 2023 Aug 18;51(4):362–3. doi: 10.4274/TJAR.2023.221172 (PMC10440487; doi:10.4274/TJAR.2023.221172)
Supplement: Related texts

# Reply: Response to the Letter

Editorial policy note on the use of retracted/withdrawn publications as references for citations

The use of retracted publications as references is a controversial issue in the world of medical literature.

The use of retracted/withdrawn publications hasn’t been advocated by the Turkish Journal of Anesthesiology and Reanimation as an editorial policy, unless such information as of basic science, anatomy, historical progress etc. is cited from these publications. The data and results of such articles shouldn’t be included into manuscripts that are submitted for publication.

Kind Regards,

Editorial Team

The Turkish Journal of Anesthesiology and Reanimation
